# Supplementary material for: High Risk of Sustained Ventricular Arrhythmia Recurrence After Acute Myocarditis
Source: J Clin Med. 2020 Mar 20;9(3):848. doi: 10.3390/jcm9030848 (PMC7141537; doi:10.3390/jcm9030848)
Supplement: Supplementary file 1 [file jcm-09-00848-s001.pdf]

ICD-10 Diagnostic codes

- I01.2 Acute rheumatic myocarditis
- I09.0 Rheumatic myocarditis
- I40.0 Infective myocarditis
- I40.1 Isolated myocarditis
- I40.8 Other acute myocarditis
- I40.9 Acute myocarditis, unspecified
- I41.0 Myocarditis in bacterial diseases classified elsewhere
- I41.1 Myocarditis in viral diseases classified elsewhere
- I41.2 Myocarditis in infectious and parasitic diseases classified elsewhere
- I41.8 Myocarditis in diseases classified elsewhere
- I51.4 Myocarditis, unspecified
- B33.2 Viral carditis

CCMP Procedure codes

- DELF013 Implantation of an Implantable Cardioverter-Defibrillator, with placement of a right intraventricular lead by transcutaneous venous route
- DELF020 Implantation of an Implantable Cardioverter-Defibrillator, with insertion of a right intraventricular lead and a lead into a left cardiac vein by transcutaneous venous route
- DELF014 Implantation of an Implantable Cardioverter-Defibrillator, with insertion of a right intraventricular and intraventricular lead, and a lead into a left cardiac vein by transcutaneous venous route
- DELF016 Implantation of an Implantable Cardioverter-Defibrillato rwithout atrial defibrillation, with insertion of an intraatrial lead and a right intraventricular lead by transcutaneous venous route
- DELF900 Implantation of an Implantable Cardioverter-Defibrillator with atrial defibrillation, with insertion of an intraatrial lead and a right intraventricular lead by transcutaneous venous route
- DELA004 Implantation of an Implantable Cardioverter-Defibrillator with direct access implantation of an epicardiac electrode
- DELA007 Implantation of a cardiac defibrillation generator

Supplementary Figure 1. International Classification of Diseases (ICD-10) diagnostic codes for myocarditis and Common Classification of Medical Procedures (Classification Commune des Actes Medicaux, CCAM) codes for the implant of a defibrillator.
